# Combination of tumor asphericity and an extracellular matrix-related prognostic gene signature in non-small cell lung cancer patients

**DOI:** 10.1038/s41598-023-46405-4

**Published:** 2023-11-27

**Authors:** Sebastian Zschaeck, Bertram Klinger, Jörg van den Hoff, Paulina Cegla, Ivayla Apostolova, Michael C. Kreissl, Witold Cholewiński, Emily Kukuk, Helen Strobel, Holger Amthauer, Nils Blüthgen, Daniel Zips, Frank Hofheinz

**Affiliations:** 1 Department of Radiation Oncology, Charité-Universitätsmedizin Berlin, Corporate Member of Freie Universität Berlin, Humboldt-Universität zu Berlin, and Berlin Institute of Health, Berlin, Germany; 2 Berlin Institute of Health (BIH), 10178 Berlin, Germany; 3 Computational Modelling in Medicine, Instiute of Pathology, Charité Universitätsmedizin Berlin, Charitéplatz 1, 10117 Berlin, Germany; 4 German Cancer Consortium (DKTK), Partner Site Berlin, Berlin, Germany; 5 Helmholtz-Zentrum Dresden-Rossendorf, PET Center, Institute of Radiopharmaceutical Cancer Research, Dresden, Germany; 6 Department of Nuclear Medicine, Greater Poland Cancer Centre, Poznan, Poland; 7 Department for Diagnostic and Interventional Radiology and Nuclear Medicine, University Hospital Hamburg-Eppendorf, Hamburg, Germany; 8 Division of Nuclear Medicine, Department of Radiology and Nuclear Medicine, Otto Von Guericke University, Magdeburg, Germany; 9 Department of Nuclear Medicine, Charité-Universitätsmedizin Berlin, Corporate Member of Freie Universität Berlin and Humboldt-Universität zu Berlin, Augustenburger Platz 1, 13353 Berlin, Germany

**Keywords:** Cancer imaging, Cancer microenvironment, Lung cancer, Tumour biomarkers, Tumour heterogeneity

## Abstract

One important aim of precision oncology is a personalized treatment of patients. This can be achieved by various biomarkers, especially imaging parameters and gene expression signatures are commonly used. So far, combination approaches are sparse. The aim of the study was to independently validate the prognostic value of the novel positron emission tomography (PET) parameter tumor asphericity (ASP) in non small cell lung cancer (NSCLC) patients and to investigate associations between published gene expression profiles and ASP. This was a retrospective evaluation of PET imaging and gene expression data from three public databases and two institutional datasets. The whole cohort comprised 253 NSCLC patients, all treated with curative intent surgery. Clinical parameters, standard PET parameters and ASP were evaluated in all patients. Additional gene expression data were available for 120 patients. Univariate Cox regression and Kaplan–Meier analysis was performed for the primary endpoint progression-free survival (PFS) and additional endpoints. Furthermore, multivariate cox regression testing was performed including clinically significant parameters, ASP, and the extracellular matrix-related prognostic gene signature (EPPI). In the whole cohort, a significant association with PFS was observed for ASP (p < 0.001) and EPPI (p = 0.012). Upon multivariate testing, EPPI remained significantly associated with PFS (p = 0.018) in the subgroup of patients with additional gene expression data, while ASP was significantly associated with PFS in the whole cohort (p = 0.012). In stage II patients, ASP was significantly associated with PFS (p = 0.009), and a previously published cutoff value for ASP (19.5%) was successfully validated (p = 0.008). In patients with additional gene expression data, EPPI showed a significant association with PFS, too (p = 0.033). The exploratory combination of ASP and EPPI showed that the combinatory approach has potential to further improve patient stratification compared to the use of only one parameter. We report the first successful validation of EPPI and ASP in stage II NSCLC patients. The combination of both parameters seems to be a very promising approach for improvement of risk stratification in a group of patients with urgent need for a more personalized treatment approach.

## Introduction

Treatment of non-small cell lung cancer (NSCLC) has rapidly changed during the last decade. Targeted therapies and immunotherapy have shown considerable benefit in metastatic stage IV patients^1,2^. The encouraging results of the PACIFIC trial have established consolidation immunotherapy for stage III patients who received definitive chemoradiation^3,4^. Due to the aggressive course of NSCLC, several trials are investigating additional targeted or immunotherapeutic approaches even in stage I and II disease. However, patient selection in these earlier stages is pivotal, since a large number of patients do not need further therapeutic escalation or do not benefit from potentially toxic adjuvant therapies. The biggest unmet clinical need for patient stratification concerns patients with stage II disease, where general recommendations reach from observation to platinum based adjuvant treatment, but may also include targeted therapy or immunotherapy.

Tumor asphericity is a measure of spatial irregularity, the asphericity values represents the fractional increase of the considered tumor’s surface area relative to that of a sphere exhibiting the same volume. Various publications have been able to show that the asphericity (ASP) of [^18^F-]fluorodeoxyglucose (FDG) uptake within primary tumors in staging positron emission tomography (PET) scans is associated with patient outcome^5–7^. ASP cut-off values successfully stratified NSCLC patients at high or low risk for tumor progression, with a large effect size and highest clinical relevance for stage II disease^8^. Only sparse data is available for biological explanation of the observed association of ASP and patient outcome. A significant correlation with the proliferation marker Ki-67 and a trend for a correlation with the expression of the VEGF receptor have been reported in a small cohort of NSCLC patients^9^. Another study was able to show a significant association of tumor ASP and EGFR mutations. EGFR mutated tumors exhibited lower ASP values than EGFR wildtype tumors^10^. However, further insights into the relationship between molecular alterations and ASP are missing so far.

Gene expression is the cell’s central mechanism to control its cellular response and identity. Therefore signatures can be extracted from the transcriptome that encode information about the properties of the cell(s) such as pathway activity, phenotype, cell/tissue type and disease state^11–14^. Next to the present state the transcriptome also carries information about the potential of the cells to respond and thus might contain information to predict the outcome of a disease or treatment. In the past 20 years, many disease-specific gene signatures have been identified, with varying prognostic or predictive value^15^.

The aim of our study was to independently validate the prognostic value of ASP in NSCLC patients and to investigate associations between published pan-cancer or NSCLC gene expression profiles and ASP.

## Patients and methods

### Data source

Original imaging data, patient characteristics, and follow up was analyzed from two European centers and three public repositories available in The Cancer Imaging archive: NSCLC Radiogenomics, TCGA LUSC/ LUAD and CPTAC LSCC/LUAD^16–21^. Additional gene expression data was available for patients from the Radiogenomics cohort (GEO accession number GSE103584) and from the TCGA cohort (NCI Genomic Data Commons (GDC)).

All patients were treated by primary surgery.

### Image acquisition

PET CT imaging for staging was performed prior to surgery. Details of image acquisition for patients from The Cancer Imaging Archive can be found in the original publications^17,22^. Patients treated at university hospital Magdeburg, Germany were imaged on a Biograph mCT ~ 64 PET/CT (Siemens Medical Solutions Inc., Knoxville, TN, USA). Data acquisition started 65.9 ± 5.7 min (range 57.4–78.0) after injection of (234 ± 11.9) MBq ^18^F-FDG. Tomographic images were reconstructed using PSF + TOF reconstruction (2 iterations, 21 subsets). Patients treated at Greater Poland Cancer Centre, Poznan, Poland were scanned on a Gemini TF TOF PET/CT (Philips Healthcare, Best, The Netherlands). Data acquisition started 68.5 ± 11.2 min (range 51.3–85.8) after injection of ^18^F-FDG with mean activity of 373 ± 67.2 MBq. Tomographic images were reconstructed using the BLOB-OS-TF reconstruction (3 iterations, 33 subsets).

### Image analysis

The metabolically active part of the primary tumor was delineated in the PET data by an automatic algorithm based on adaptive thresholding considering the local background^23,24^. The resulting delineation was inspected visually by an experienced observer and corrected manually where this was deemed necessary. This happened in 16 of 253 cases exhibiting only low diffuse tracer accumulation in the respective lesions. For the delineated ROIs the metabolic active tumor volume (MTV), maximum standardized uptake value (SUV_max_), total lesion glycolysis (MTV x SUV_mean_, TLG), and ASP were computed, where ASP was determined as described previously^6,25^. ROI definition and analysis was performed using the ROVER software, version 3.0.51 (ABX, Radeberg, Germany).

### Gene expression analysis

Various previously published gene expression signatures that have been proposed as pan-cancer signatures or in NSCLC were investigated. These signatures included hypoxia, extracellular matrix-related genes, immunotherapy related signatures and others. Details on the respective signatures can be found in the original publications^26–35^.

Raw reads for the radiogenomics data set were downloaded from the Sequence Read Archive (SRA, project number PRJNA401995). Reads were mapped to human reference genome GRCh38.p13 using STAR aligner v2.7.9c with GENCODE v38 annotation^36^. Aligned reads were quantified using featureCounts from subread v2.0.1.

Count data for the TCGA data set were downloaded using the R package TCGAbiolinks v2.25.3^37^. Both data sets were filtered for genes with insufficient counts (> 1 count in whole data set) and subsequently normalized using the R package DESeq2 v1.37.6^38^.

### Gene signatures

Genes of the following signatures were used in this study (Supplemental Fig. 1):

**Table:** 

Signature	Abbreviation	# Genes	Source
Tumor inflammation signature	TIS	18	^28^
T-effector and interferon-γ gene signature	TEffInfG	8	^32^
Radiosensitivity index genes	RadSensInd	10	^27^
T cell-inflamed Gene Expression Profile signature	TCellInfGEP	18	^30^
Immune signature for LUAD prognosis	ImmLUADProgn	18	^31^
Response to MAGE-A3 immunotherapeutic	MAGA3ImmTher	91	^33^
Common hypoxia metagene	HypoxMeta	15	^29^
ECM-related prognostic and predictive indicator	EPPI	29	^26^
Hypoxia-induced EMT	HypoxEMT	17	^34^
Small extracellular vesicle-associated signature	ExtraCellVes	15	^35^

EPPI risk score (in this paper abbreviated as EPPI) was calculated using the original formular and coefficients published previously^26^.

To investigate if PET parameters and information of prognostic or predictive gene signatures are correlated with each other, several gene sets that have been investigated in NSCLC patients or as pan-cancer signatures were calculated in a sub-group of patients (radiogenomics and TCGA cohort). To see if the observed correlations are reproducible, both cohorts were investigated separately. The only gene set that showed a reproducible weak negative correlation with ASP in both cohorts was the extracellular matrix-related prognostic and predictive indicator (EPPI), published by Lim and colleagues (Supplementary Fig. 1).

### Statistical analysis

Primary clinical endpoint was progression free survival (PFS), defined as absence of occurrence of any disease recurrence (loco-regional or distant) or death. In addition, loco-regional tumor control (LRC), freedom from distant metastases (FFDM), and overall survival (OS) measured from the date of surgery to death and/or event, were analyzed. Patients who did not keep follow-up appointments and for whom information on survival or tumor status therefore was unavailable were censored at the date of last follow-up.

The association of OS, LRC, FFDM, and PFS with clinically relevant parameters (sex, age, histology, T-stage, N-stage, and UICC-stage) as well as quantitative PET parameters and gene signatures was analyzed using univariate Cox proportional hazard regression in which the PET parameters were included as metric and as binarized parameters, respectively. The cutoff values used for binarization were calculated by performing a univariate Cox regression for each measured value. The values leading to the hazard ratio (HR) with the highest significance were used as cutoff. To avoid too small group sizes, only values within the interquartile range were considered as potential cutoff. Cutoff values were separately computed for OS, LRC, FFDM, and PFS. For validation of ASP, a previously published cutoff was applied without using the cutoff optimization method described here.

The probability of survival was computed and rendered as Kaplan–Meier curves. Independence of parameters was analyzed by multivariate Cox regression.

Statistical significance was assumed at a P-value of less than 0.05. Statistical analysis was performed with the *R language and environment for statistical computing* version 4.2.1^39^.

## Results

**Figure 1:**
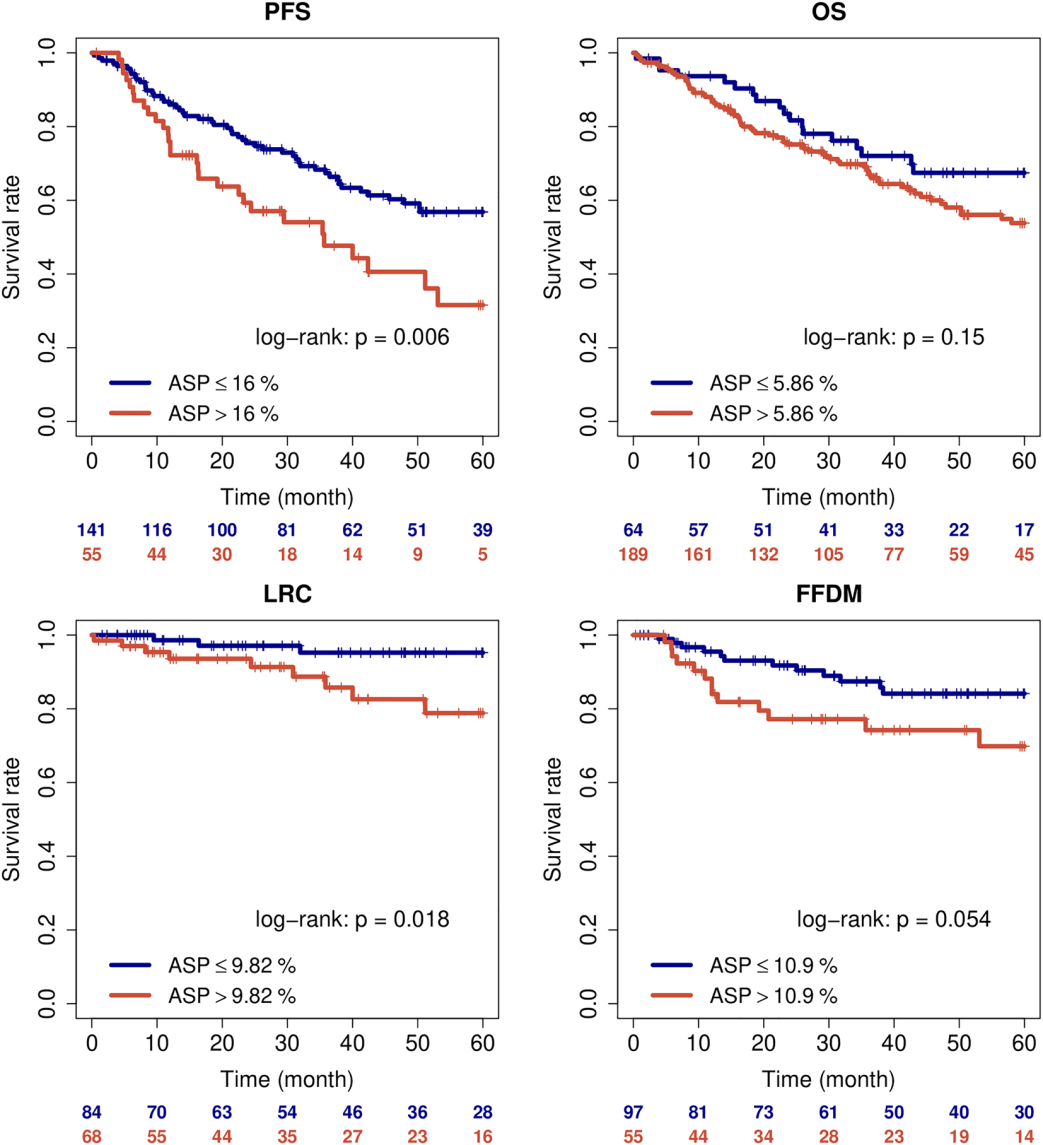
Kaplan Meier curves of all surgically treated patients stratified according to tumor asphericity.

253 patients with NSCLC and curative intended surgery were analyzed. Most patients were male and UICC stage I. Univariate cox regression revealed a significant association of ASP with PFS and a trend for OS (Table 1). With binarized cut-off values, ASP significantly discriminated between high and low risk patients for the investigated endpoints PFS and LRC and showed a trend for FFDM (see Kaplan Meier plots Fig. 1 and Supplementary Table 1). Other PET parameters with significant association with outcome were, both, SUV_max_ and MTV, showed an association with LRC. SUV_max_ also significant a association with PFS.

**Table 1:** Univariate cox regression analysis, all PET parameters were included as metric parameters.

Parameter	PFS				OS			
	N	HR	95% CI	P-value	N	HR	95% CI	P-value
Sex male	196	0.95	0.59–1.5	0.82	253	1.18	0.76–1.84	0.45
Age > 70y	196	1.37	0.88–2.12	0.16	196	1.58	1.04–2.42	**0.034**
T-stage > 2	190	0.84	0.47–1.5	0.55	247	0.8	0.46–1.39	0.43
N-stage > 0	196	2.47	1.56–3.91	< **0.001**	253	2.45	1.59–3.76	< **0.001**
UICC-stage > II	196	2.03	1.23–3.35	**0.0054**	253	2.05	1.3–3.25	**0.002**
Histology SCC	188	1.61	1.01–2.55	**0.044**	188	1.52	0.89–2.61	0.12
EPPI score > 8.72	120	4.46	1.39–14.36	**0.012**	157	2.4	0.95–6.05	**0.063**
MTV	196	1.003	0.998–1.008	0.19	253	0.998	0.991–1.005	0.58
TLG	193	1	1–1	0.43	249	1	0.999–1	0.3
SUVmax	193	1.04	1.01–1.06	**0.0067**	249	1.01	0.99–1.04	0.29
ASP	196	1.02	1.01–1.03	< **0.001**	253	1.01	1–1.02	**0.071**

Parameter	LRC				FFDM			
	N	HR	95% CI	P-value	N	HR	95% CI	P-value
Sex male	152	1.01	0.3–3.36	0.99	152	0.67	0.3–1.48	0.32
Age > 70y	152	0.79	0.24–2.62	0.7	152	1.22	0.55–2.68	0.63
T-stage > 2	146	1.8	0.48–6.78	0.39	146	1.52	0.61–3.8	0.37
N-stage > 0	152	2.94	0.88–9.84	**0.08**	152	4.46	2.02–9.86	< **0.001**
UICC-stage > II	152	3.54	1.06–11.8	**0.039**	152	3.98	1.76–9.02	< **0.001**
Histology SCC	152	1.74	0.52–5.77	0.37	152	0.88	0.33–2.35	0.8
EPPI score > 8.72	120	2.66	0.33–21.06	0.36	120	–	–	–
MTV	152	1.009	1–1.017	**0.042**	152	1.005	0.997–1.013	0.24
TLG	150	1	1–1.001	0.21	150	1	1–1.001	0.23
SUVmax	150	1.06	1–1.13	**0.035**	150	1.05	1–1.1	**0.052**
ASP	152	1.02	0.98–1.06	0.32	152	1.02	0.99–1.04	0.15

P-values of significant parameters and of parameters showing a trend for significance are in bold.

**Figure 2:**
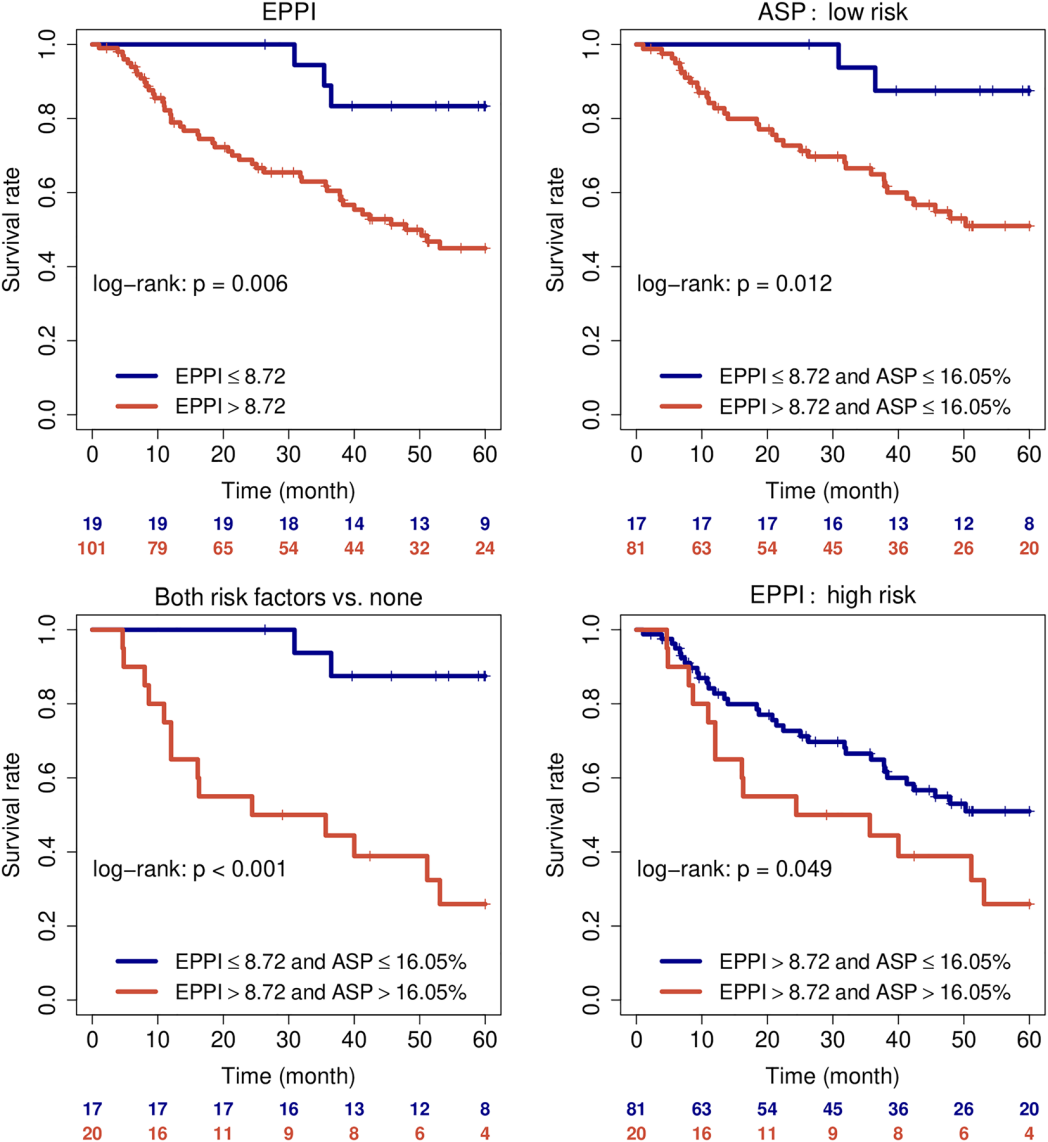
Progression free survival of patients when stratified according to EPPI for all patients (**A**) and for patients stratified by PET measured ASP as low risk group (**B**). Additional stratification using combined risk factors (PET asphericity and EPPI risk score (**C**) and additional stratification benefit of patients stratified by EPPI risk score as high-risk with additional PET ASP information (**D**).

Since the EPPI risk score was originally established using the TCGA database, we used patients of the radiogenomics cohort only to see if this signature can be validated independently. In this subgroup of 120 patients it was possible to validate the prognostic impact of the signature by univariate cox regression analyses (Table 1). Furthermore, the combined information of EPPI and PET measured tumor asphericity seems to provide additional prognostic information as shown by the Kaplan Meier plots in Fig. 2. Multivariate testing of ASP, EPPI risk score, and clinical parameters revealed a significant association of ASP in the whole cohort and a significant association of EPPI in the sub-group of patients with gene expression data. The results of multivariate testing are shown in Table 2. As a side note, SUV_max_ showed similar association with outcome upon multivariate testing (Supplementary Table 2).

**Table 2:** Multivariate cox regression analysis, ASP and EPPI were included as metric parameters. Results are shown for the whole cohort (n = 188) and for the radiogenomics cohort with gene expression data (n = 120).

Parameter	HR	95% CI	P-value
PFS (N = 188)			
UICC-stage	1.51	0.872–2.6	0.14
Histology	0.949	0.777–1.16	0.6
ASP	1.02	1.0–1.03	**0.012**
PFS (N = 120)			
UICC-stage	2.21	1.15–4.25	**0.018**
Histology	1.2	0.882–1.64	0.24
EPPI score	4.14	1.27–13.5	**0.018**
ASP	1.01	0.995–1.03	0.15

P-values of significant parameters and of parameters showing a trend for significance are in bold.

**Figure 3:**
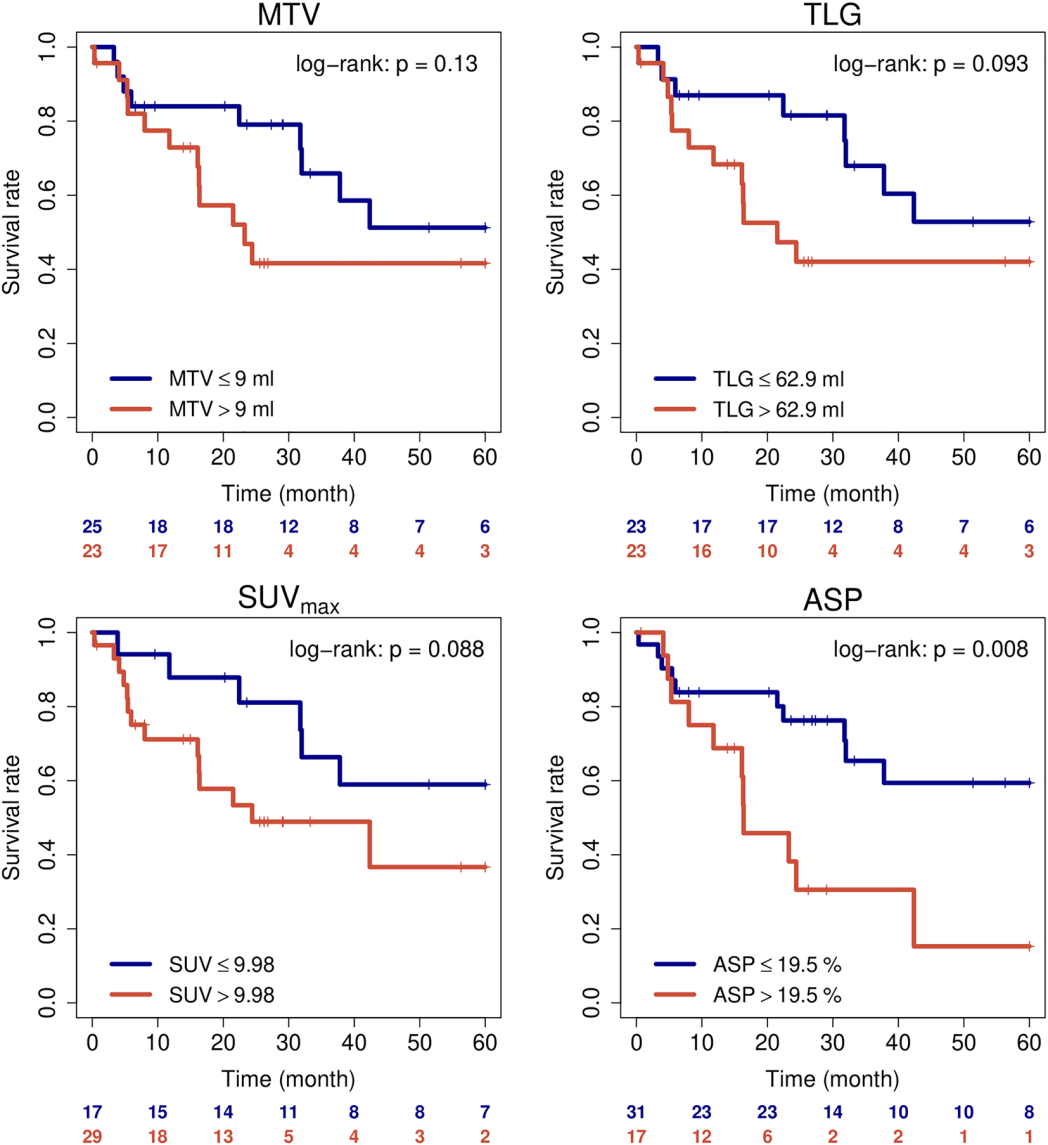
Kaplan Meier curves showing progression-free survival of all surgically treated UICC stage II patients stratified according to PET parameters. For each PET parameters the best cut-off value was applied, except ASP, here a previously published cut-off value was applied.

Since both EPPI and ASP were initially evaluated in stage II disease, further analysis was restricted to this tumor stage. In stage II patients, ASP was the only PET parameter that was significantly associated with PFS (Table 3). Furthermore, after binarization using previously published cut-off values, ASP significantly discriminated low and high risk stage II patients, while other PET parameters did not show a significant discrimination between risk groups, despite cut-off optimization (Supplementary Table 3). Figure 3 shows Kaplan Meier plots for the previously published ASP cutoff and routinely used PET parameters.

**Table 3:** Univariate cox regression analysis for UICC stage II patient. All PET parameters were included as metric parameters.

Parameter	PFS				OS			
	N	HR	95% CI	P-value	N	HR	95% CI	P-value
Sex male	48	1.01	0.41–2.52	0.98	65	2.88	0.98–8.49	**0.055**
Age > 71y	48	2.37	1–5.63	**0.049**	48	2.14	0.92–4.95	**0.076**
T-stage > 2	48	0.45	0.19–1.1	**0.081**	65	0.36	0.14–0.91	**0.032**
N-stage > 0	48	1.82	0.77–4.29	0.17	65	1.56	0.69–3.54	0.29
Histology SCC	43	2.5	1.03–6.05	**0.043**	43	1.44	0.52–3.99	0.48
EPPI score > 21.3	27	3.83	1.12–13.13	**0.033**	39	2.56	0.86–7.62	**0.091**
MTV	48	1.003	0.995–1.011	0.47	65	0.997	0.986–1.008	0.62
TLG	46	1	0.999–1.001	0.7	62	1	0.999–1.001	0.55
SUVmax	46	1.03	0.98–1.09	0.24	62	1.01	0.96–1.07	0.61
ASP	48	1.02	1–1.03	**0.0087**	65	1.01	1–1.03	**0.068**

Parameter	LRC				FFDM			
	N	HR	95% CI	P-value	N	HR	95% CI	P-value
Sex male	32	–	–	–	32	–	–	–
Age > 71y	32	2.04	0.17–24.8	0.58	32	0.89	0.09–8.86	0.92
T-stage > 2	32	1.41	0.13–15.87	0.78	32	0.28	0.03–2.71	0.27
N-stage > 0	32	1.04	0.09–11.5	0.98	32	5.66	0.58–54.79	0.13
Histology SCC	32	4.36	0.39–48.2	0.23	32	0.72	0.07–6.95	0.78
EPPI score > 21.3	27	–	–	–	27	1.81	0.18–17.85	0.61
MTV	32	1.01	1–1.03	**0.037**	32	0.96	0.87–1.06	0.41
TLG	31	1.002	1–1.003	**0.033**	31	0.997	0.988–1.006	0.45
SUVmax	31	1.08	0.95–1.22	0.25	31	1.04	0.93–1.17	0.47
ASP	32	1.006	0.934–1.085	0.87	32	0.99	0.91–1.07	0.76

P-values of significant parameters and of parameters showing a trend for significance are in bold.

**Figure 4:**
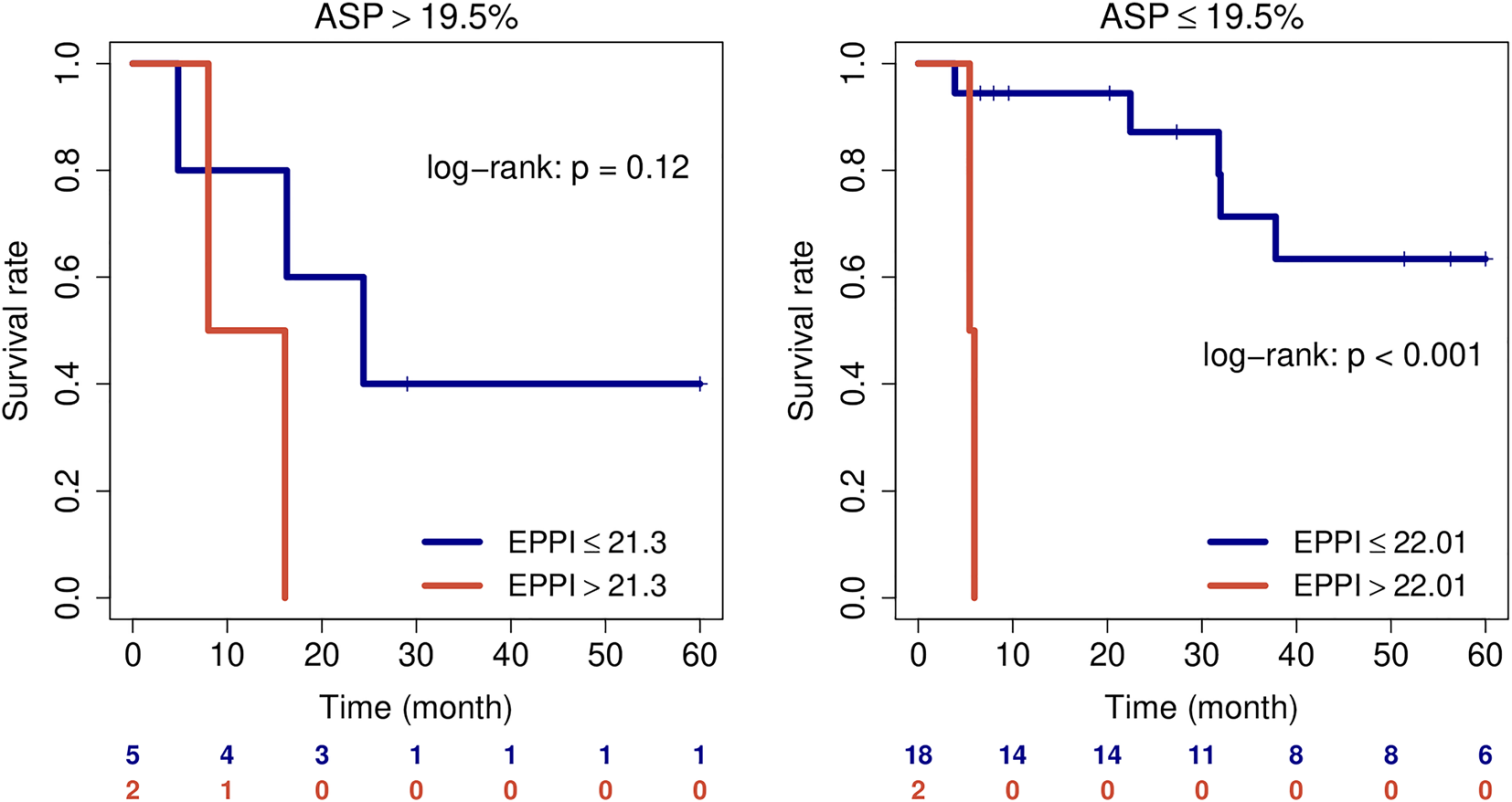
Patient stratification by EPPI in UICC II patients of the radiogenomics cohort that have been stratified by the PET parameter ASP into high risk and low risk groups.

In the radiogenomics sub-group of patients with PET and genomic data, the incremental value of additional gene expression information was investigated. Patients stratified according to ASP were further stratified based on their EPPI. This analysis revealed that EPPI seems to have an additional prognostic value, especially in low risk patients according to ASP, where EPPI classification significantly improved risk stratification as shown in Fig. 4. Multivariate testing of clinical parameters, ASP, and EPPI in stage II patients revealed EPPI risk score as the only significant parameter as depicted in Table 4. However, this finding has to be interpreted cautiously due to the low number of only 27 patients in this sub-group. When analyzing all patients with imaging data, ASP remained significantly associated with PFS, as shown in Table 4.

**Table 4:** Multivariate cox regression analysis for UICC stage II patients, either for patients with gene expression data (n = 27) or for all patients with imaging data (n = 43 and n = 65 for OS, respectively).

Parameter	HR	95% CI	P-value
PFS (N = 27)			
Histology	1.91	0.385–9.5	0.43
EPPI score	5.72	1.16–28.2	**0.032**
ASP	1.01	0.984–1.04	0.37
PFS (N = 43)			
Age	2.11	0.853–5.2	0.11
Histology	0.462	0.188–1.13	**0.092**
ASP	1.02	1–1.03	**0.015**
OS (N = 65)			
Sex	3.32	1.1–9.98	**0.033**
Age	1.83	0.769–4.33	0.17
T-stage	0.314	0.12–0.82	**0.018**
ASP	1.01	1–1.03	**0.057**

P-values of significant parameters and of parameters showing a trend for significance are in bold.

## Discussion

We independently validated the novel PET parameter tumor asphericity and the extracellular matrix-related prognostic gene expression signature in NSCLC patients. Both parameters were significantly associated with progression-free survival of surgically treated patients and independent from established clinical parameters. ASP has previously shown most promising stratification potential in stage II patients and we were able to independently validate the published cut-off value in this group. Even EPPI risk score was initially investigated for stage I and II disease and the strong prognostic value of EPPI risk score could successfully be validated in the whole group, but also in stage II patients alone. Most importantly the combination of EPPI risk score and ASP seems to improve patient stratification compared to the use of only one biomarker. This result has to be interpreted cautiously due to the relatively low number of patients with stage II disease and gene expression data. Nonetheless, the observed clinical effect is strong and affects a group of NSCLC patients with an urgent need for more personalized treatment approaches. Recommendations for resected stage II patients include no further treatment or adjuvant platinum based chemotherapy. Additionally, a recently published phase III trial compared the checkpoint-inhibitor pembrolizumab with placebo in resected stage IB-IIIA NSCLC patients^40^. Additionally, the IMpower010 trial investigated the use of the checkpoint inhibitor atezolizumab in a similar setting and observed a disease-free survival benefit with atezolizumab versus best supportive care after adjuvant chemotherapy in patients with resected stage II–IIIA NSCLC^41^. Nevertheless, one has to bear in mind that median progression free survival in the trial cohort was about three years in the control arm and the effect of adjuvant pembrolizumab was moderate in the whole study population. Given the relevant toxicities of chemotherapy and immunotherapy, an improvement of patient risk stratification is urgently needed. The combination of gene and imaging data seems a very promising approach. Conventional gene expression data, especially if performed on biopsy specimens, is not able to reflect intratumor heterogeneity and characteristics of the tumor micromilieu. Single-cell profiling experiments have shown a high genetic heterogeneity in NSCLC^42^. Due to the complexity of single-cell analyses and high-costs, this approach is not affordable on a large scale, yet. PET imaging might therefore be a well-suited modality to assess individual tumor heterogeneity as a complement to conventional gene expression analyses, as shown by our study.

Data on the combined use of gene expression and PET imaging data is sparse and most of the published data has only exploratory character. One publication evaluated the combined use of PET parameters and Thymidylate Synthase Expression in stage IV NSCLC patients. In this group of patients, the authors found a significant correlation of Thymidylate Synthase Expression and TLG. TLG showed the most promising prognostic value regarding several important outcome measures including PFS. However, there did not seem to be added value by the combined use of both parameters^43^. In patients treated with curative intent, a recent analyses evaluated PET, CT, and genome data of surgically resected NSCLC patients^44^. In this study PET and CT information failed to predict disease recurrence but some gene expression profiles seemed deliver helpful information regarding disease recurrence. Nonetheless, the study was a retrospective single center analyses that would need further validation. Another interesting study used TCGA and institutional data to develop a PET CT radiomics signature that can predict tumor immune profiles in NSCLC. The resulting model could be successfully validated in an independent dataset and is therefore a promising predictive biomarker for immunotherapies^45^. Two other publications have investigated the combined use of tumor or circulating immune parameters and PET parameters of resected NSCLC patients^46,47^. Both publications reported some correlation with immune parameters and an association of PET parameters with clinical outcome. These data are interesting because adjuvant checkpoint inhibition would be a reasonable therapy for selective patients in case of a good predictive marker. Nonetheless, both publications only investigated very basic PET parameters like SUV_max_ and TLG. Additionally, the observed effect size was not that large and these parameters do not seem to be optimal for individual treatment personalization.

A big strength of our study is the independent validation of both, the ASP PET parameter and the EPPI gene expression signature. The additional finding that these biomarkers are not strongly correlated with each other and potentially provide independent prognostic value indicate that these biomarkers merit further prospective validation, which is a prerequisite for future clinical use. The retrospective nature of the current analyses is a major limitation due to the well-known shortcomings of retrospective data. Additionally, while the multicentric nature of our analyses is principally advantageous in terms of future application in a multicenter setting, it also can be viewed as a limitation: while all patients received surgical treatment, surgical techniques changed over time and might potentially affect outcome of patients. Furthermore, reconstruction algorithms have been shown to affect ASP^48^. Although in most cases discrepancies were only moderate and can potentially be addressed by smoothing/ re-scaling the data, this has not been investigated in the current analysis.

Overall, our analysis independently validates the strong prognostic value of ASP and shows great potential for treatment personalization when combined with EPPI risk score.

### Supplementary Information

Supplementary Information.

## Data Availability

The datasets generated and analysed during the current study are available in the Synapse repository, http://www.synapse.org with the synID syn52673953.
