# Supplementary material for: Combination of tumor asphericity and an extracellular matrix-related prognostic gene signature in non-small cell lung cancer patients
Source: Sci Rep. 2023 Nov 27;13:20840. doi: 10.1038/s41598-023-46405-4 (PMC10681996; doi:10.1038/s41598-023-46405-4)
Supplement: Supplementary file 1 — Supplementary Information. [file 41598_2023_46405_MOESM1_ESM.docx]

**Supplementary Figure 1:** Correlation of published gene signatures with tumor asphericity for the radiogenomics (n = 120) and TCGA (n = 37) cohort. Gene expression of each individual gene was correlated (Spearman) with the respective ASP-values in that cohort and the correlations of genes belonging to a signature were summarized in a boxplot (median correlation indicated numerically). Note that not each gene was present in each data set (# genes in data, bottom). Significance was assessed by a permutation test using the perm.cor.test function from the R package jmuOutlier (fraction with p<0.05), top).

**Supplementary Figure 2:** Correlation of individual EPPI genes with ASP within the TCGA cohort

**Supplementary Figure 3:** Correlation of individual EPPI genes with ASP within the Radiogenomics cohort

**Supplementary table 1:** Univariate Cox regression analyses. PET parameters were included as binarized parameters.

**Supplementary table 2:** Multivariate cox regression analysis, SUV_max_ and EPPI were included as metric parameters. Results are shown for the whole cohort (n =186) and for the radiogenomics cohort with gene expression data (n = 118).

**Supplementary table 3:** Univariate Cox regression analyses for stage II patients. PET parameters were included as binarized parameters.
